# Procedure for a standardized preparation of skin prick test solutions for the diagnosis of occupational type I allergies in the absence of commercial extracts 

**DOI:** 10.5414/ALX02506E

**Published:** 2024-07-04

**Authors:** Sabine Kespohl, Robin Jost, Silke Maryska, Lena-Maria Altin, Ingrid Sander, Stefan Schülke, Kathrin E. Paulus-Tremel, Andreas Bonertz, Thomas Klose, Vera Mahler, Monika Raulf

**Affiliations:** 1 Institute for Prevention and Occupational Medicine of the DGUV, Ruhr-University Bochum (IPA), Bochum,; 2 Division Allergology, Paul-Ehrlich-Institut, Langen, and; 3 Sonnenschein Apotheke, Koblenz, Germany; * Current address: University Bayreuth, Kulmbach, Germany

**Keywords:** allergy diagnosis, occupational allergens, skin prick test solutions, quality, standardization, diagnostic gap, public pharmacy, test allergen preparation

## Abstract

In order to ensure valid diagnostics for occupational test allergen solutions despite the ongoing reduction in the availability of commercial test extracts, a plan B was initiated for the possible production of skin prick test (SPT) solutions in public pharmacies. For important occupational allergen sources (wheat and rye, storage mites, animal epithelia, mold material) laboratory extraction methods were analyzed in comparison to pharmacy compatible extraction methods regarding protein quantity and quality in SDS-PAGE combined with silver staining. Subsequently, using the example of bovine epithelia, adapted extraction procedures as well as in-process and final product controls were transferred to a public pharmacy. Allergen sources with a high protein content, such as wheat and rye grains as well as storage mites, showed good comparability of the extractable protein quantity and protein pattern, regardless of the applied extraction method. In contrast, allergen source materials with a low total protein content, such as animal epithelia and molds, can benefit from laboratory extraction conditions such as mechanical disruption and specific buffer additives. In the qualitative protein silver staining, characteristic protein patterns were identified for each allergen source. Depending on the extraction method, only minor differences in total protein patterns were observed in animal epithelia and molds. Using source materials from two suppliers, the resulting allergen extracts displayed clear differences in protein content in storage mites and quantitative and qualitative differences in molds. A practical preparation attempt of SPT solutions in a public pharmacy was successful. SPT solutions prepared with adapted pharmacy extraction methods showed a comparable protein and Bos d 2 allergen content and equivalent qualities in the protein pattern compared to a previously available commercial SPT solution. Accordingly, it can be assumed that standardized SPT solutions with sufficient allergen quality for occupational allergen sources can be prepared in public pharmacies if certified allergen sources with appropriate protein content are available.

## Introduction 

Occupational exposure to sensitizing substances can lead to allergic diseases of the upper and lower respiratory tract or the skin. Currently, more than 400 occupational substances are documented as potentially IgE-sensitizing, and new ones are added every year. This increase is paralleled by a simultaneous reduction in commercially available allergen extracts for skin and provocation tests (see contribution by Zimmer & Mahler in Allergologie 2024 [1]). According to the German statistics for occupational diseases, a total of 2,861 cases of obstructive respiratory diseases caused by sensitizing substances, including rhinopathy (occupational diseases (BK) number 4301), were recognized during 2018 – 2022. Flour and bakery products were assessed as the triggering substance in 69% of cases, followed by animal hair and epithelia in 12.5%, dust from food and animal feed (fish, seafood, mites) in 3.3%, and molds in 2.2% of cases [2]. The typical step-by-step diagnosis in case of suspected type I allergy starts with a detailed medical history, followed by skin prick tests (SPTs), in-vitro tests and, if necessary, a specific provocation test. For occupational type I allergies, the workplace inhalation challenge was described as the gold standard [3], if conducted in a standardized and qualified manner. The indication must be critically examined beforehand, and an acceptable health risk for the patient must be considered. Alternatively, for high molecular weight allergens like wheat and rye flour, it has been shown that a positive skin test and/or the detection of specific IgE can be used to predict the outcome of specific challenge tests with flours in symptomatic bakers [4, 5]. Furthermore, the SPT is a quick, cost-efficient, and sensitive method for detecting type I sensitization. It can be carried out in any medical practice; the test result is immediately visible and has an informative effect for the patient. Therefore, SPTs are the most frequently applied diagnostic tool in the EU for ~ 90% of suspected cases of type I allergic disease of the respiratory tract (rhinitis and asthma) [6]. However, SPT allergens are medicinal products with corresponding requirements, e.g., they must obtain a marketing authorization and each batch has to undergo batch release testing to ensure consistent quality [1, 7]. These cost-inherent procedures in combination with a low demand influence the availability of test allergens in particular for rare allergen sources. A call for action was published as an EAACI position paper [8], which warns of a trend reversal in modern allergy diagnostics towards non-standardized, individual allergen tests. This completely contradicts the medical need to work with safe and effective allergen solutions of proven quality for in-vivo allergy diagnostics. To counteract this situation, a research project was initiated by the German Social Accident Insurance with the aim to improve the long-term availably of standardized allergy test instruments for occupational allergic diseases to ensure a reliable diagnosis of type I allergy. For this purpose, the 20 most relevant occupational allergens were initially agreed with the accident insurance institutions [2]. In order to close the diagnostic gap, it was investigated whether a production of occupational test allergens in public pharmacies might be possible in accordance with the German Medicinal Products Act and considering the European Pharmacopoeia and further legal aspects, as described in [7]. The following article reports on allergen extraction procedures for occupational allergens under laboratory conditions in comparison to pharmacy conditions and presents the transfer attempt of the extraction procedures to a public pharmacy using bovine epithelia as an example. 

## Materials and methods 

**Figure 1:**
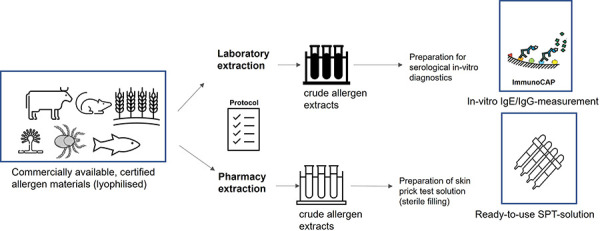
Schematic diagram of allergen extractions under standard laboratory conditions versus pharmacy conditions and preparation of skin prick test solutions.

Based on the priority list for occupational allergens, lyophilized allergen materials were purchased from the certified allergen suppliers Allergon AB (Ängelholm, Sweden) and Stallergenes Greer (Lenoir, CA, USA) and tested for different extraction methods (Figure 1). Quantities of allergen material (Table 1) were weighed and extracted in a slightly modified manner according to establishedlaboratory extraction protocols for in-vitro IgE/IgG diagnostics: wheat and rye flours [9], storage mites [10], animal epithelia [11], and molds [12]. On the other hand, an extraction protocol with reduced requirement of specialized laboratory equipment was used for the adapted preparation in a public pharmacy. Details of both extraction methods are described below. 

### Allergen extraction under laboratory conditions 

**Extraction buffers**

For wheat and rye grains, a neutral extraction with potassium phosphate buffer (10 mM K_2_PO_4_, 400 mM NaCl, titrated with 10 mM KPO_4_×2H_2_O, 400 mM NaCl adjusted to pH 7.0) was performed first, followed by ethanolic extraction using 70% ethanol (Rotipuran ≥ 99.8% p.a., Carl Roth, Karlsruhe, Germany) diluted in A. bidest. 

For all other allergens, phosphate buffered saline (PBS) (137 mM NaCl, 1.47 mM KH_2_PO_4_, 8.1 mM Na_2_HPO_4_×2H_2_O, 2.7 mM KCl; pH 7.4) with 1 mM Pefabloc (animal epithelia and mites) or 10 mM Pefabloc (molds) was used. 

**Extraction procedures**

Lyophilized allergen materials were suspended in extraction buffers according to Table 1 and incubated for 10 minutes at room temperature by roll mixing, at 36 rounds per minute (Tilt/roll mixer RS-TR 10, Phoenix Instrument, Garbsen, Germany). Afterwards, different mechanical treatments were applied for cell disruption. 

Wheat and rye grain material was extracted in two steps. First, neutral extraction was performed by incubating the lyophilized material in extraction buffer at 4 °C for 2 hours on a roll mixer at 36 rounds per minute. Insoluble particles were sedimented by centrifugation for 30 minutes at 20,000 g at 4 °C. The clear supernatant was dialyzed against 5 mM potassium phosphate buffer, pH 7, in a dialysis cassette, exclusion size 3.5 kDa (Slide A Lyzer, ThermoFisher Scientific, Waltham, MA USA) according to manufacturer’s instructions. Secondly, the sediments of the neutral wheat and rye extraction were used for additional ethanolic extraction. They were resuspended in half of the original extraction volume in 70% ethanol, vortexed, and mixed for 1 hour at 4 °C, 36 rounds per minute on a roll mixer. Subsequently, insoluble particles were sedimented as described above, and ethanolic extracts were dialyzed against 1% acetic acid in a dialysis cassette, exclusion size 3.5 kDa. Precipitated proteins after dialysis in neutral and ethanolic extracts were sedimented (30 minutes, 20,000 g, 4 °C), and clear supernatants were used for further processing. 

Storage mite and animal epithelia material was homogenized in 10 mL cylindrical or conical homogenizers (Schuett Biotec, Göttingen, Germany) for 5 minutes at 2,000 rounds per minute using Schuett homogen plus as homogenizer drive, cooled with iced water. The allergen suspension was transferred into a 5-mL Eppendorf tube and sonicated (Sonorex Super RK 255, Bandelin, Germany) in iced water for 10 minutes. Insoluble particles were sedimented by centrifugation for 30 minutes at 20,000 g at 4 °C. 

Mold material was homogenized with an SK38 glass/ceramic bead mixture in Precellys24 (PeqLab, Erlangen, Germany) for 3 cycles of 20 seconds with 30-second breaks between each cycle at 6,000 agitation per minute at 4 °C. The extracts were then incubated in an ultrasonic bath with iced water for 10 minutes. Beads and cell debris were sedimented by centrifugation at 2,820 g at 4 °C for 5 minutes. The clear supernatant was transferred into a new tube and centrifuged again at 20,000 g at 4 °C for 45 minutes. 

### Allergen extraction adapted to pharmacy conditions 

**Extraction buffers**

For all allergen materials including neutral extraction of wheat and rye grains, sterile 0.9% NaCl (B. Braun SE, Melsingen, Germany) was used as extraction buffer. For ethanolic extraction of wheat and rye allergens, 70% ethanol (Rotipuran ≥ 99.8% p.a.) diluted with 0.9% NaCl was used. 

**Extraction procedures**

Lyophilized allergen materials, including wheat and rye (neutral extraction), were suspended in 0.9% NaCl according to Table 1 and incubated for 2 hours at room temperature by end-over-end rotation (multi-axial mixer, rotator, VWR International, Darmstadt, Germany) at 18 rounds per minute. For ethanolic extraction of wheat and rye, sediments of neutral extraction were resuspended in half of the original extraction volume in 70% ethanol/0.9% NaCl and incubated for 1 hour by end-over-end rotation, 18 rounds per minute, at room temperature. The wheat and rye extracts were not dialyzed. 

**Preparation of sterile SPT solutions**

SPT solutions from allergen extracts were prepared in 0.9% NaCl under pharmacy conditions and filtered with a 0.45-µm syringe filter (Chromafil CA-45/25 (S), Macherey-Nagel, Düren, Germany). SPT solutions were prepared as 50% (v/v) glycerol solutions, therefore 1.26 g (equals 1 mL) glycerol (99.9%, Caelo, Hilden, Germany) were weighed per 1 mL allergen extract and mixed to homogeneity. The allergen solution was drawn into a sterile 10-mL syringe (Omnifix 10 mL, B. Braun SE, Melsingen, Germany) and transferred via a 0.22-µm filter unit (Millex-GP Syringe Filter Unit, PES, 33 mm, 0.22 µm, Millipore, Merck, Darmstadt, Germany) with a sterile Luer lock connection to a sterile 1-mL dosing syringe (Omnifix 1 mL, B. Braun SE). 

**Transfer of the extraction procedure of bovine epithelial allergens to a public pharmacy**

The laboratory equipment required for the mechanical cell disruption of bovine epithelia (see above) was installed in a pharmacy. In addition, pharmacy-specific equipment such as an ointment mixing device (Topitec, WEPA, Hillscheid, Germany) was used for the extraction of bovine epithelia. The extraction was always carried out in 0.9% NaCl as described above with modifications according to Table 2A. The pH value (universal pH 0 – 14 indicator strips) and the color of the extracts were documented as in-process controls. 

**Determination of protein concentration**

Protein concentration was measured in all prepared extracts using the Bradford assay (Bio-Rad, Munich, Germany) with bovine serum albumin as reference standard according to the manufacturer’s instructions. 

**SDS-PAGE and protein silver staining**

Extracts of different allergen materials were separated by sodium dodecyl sulfate – polyacrylamid gel electrophoresis (SDS-PAGE) using pre-cast 10% Bis-Tris NuPAGE gels (Invitrogen, San Diego, CA, USA) and the Xcell II mini-cell unit of Novex (Invitrogen) for 42 minutes at 200 V, according to the manufacturers’ instructions. An unstained SDS-PAGE protein marker from 6.5 – 97 kDa (Serva, Heidelberg, Germany) was used to calculate molecular weights. The same sample volume per lane was loaded for all allergen extracts (except the allergen extracts of wheat and rye grain, which were diluted 1 : 10 or 1 : 20, respectively) (Figure 4). Allergen extract (30 µL) was mixed with 10 µL SDS sample buffer (Laemmli), boiled (5 minutes at 95 °C), and subjected to SDS-PAGE. Into each gel pocket, 20 µL of boiled protein sample was loaded, resulting in final protein amounts of at least 0.3 µg and a maximum of 8 µg/lane. 

Nonquantitative silver staining was conducted with slight modification according to [13] as described in [12]. 

**Determination of Bos d 2 content in SPT solutions**

Allergen concentration (Bos d 2) was measured in all cattle epithelia SPT solutions prepared in a public pharmacy as well as in a previously available SPT solution of a commercial supplier, using the Bos d 2 ELISA kit (Indoor Biotechnologies, Manchester, UK) according to manufacturer’s instructions. 

## Results 

### Influence of allergen source material and extraction method on total protein quantity in allergen extracts 

**Figure 2:**
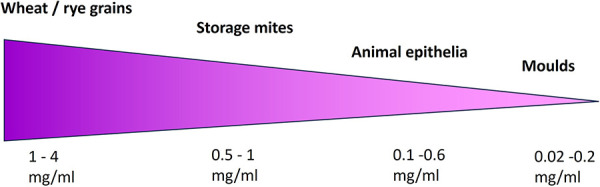
Overview of protein content of allergen source materials.

**Figure 3:**
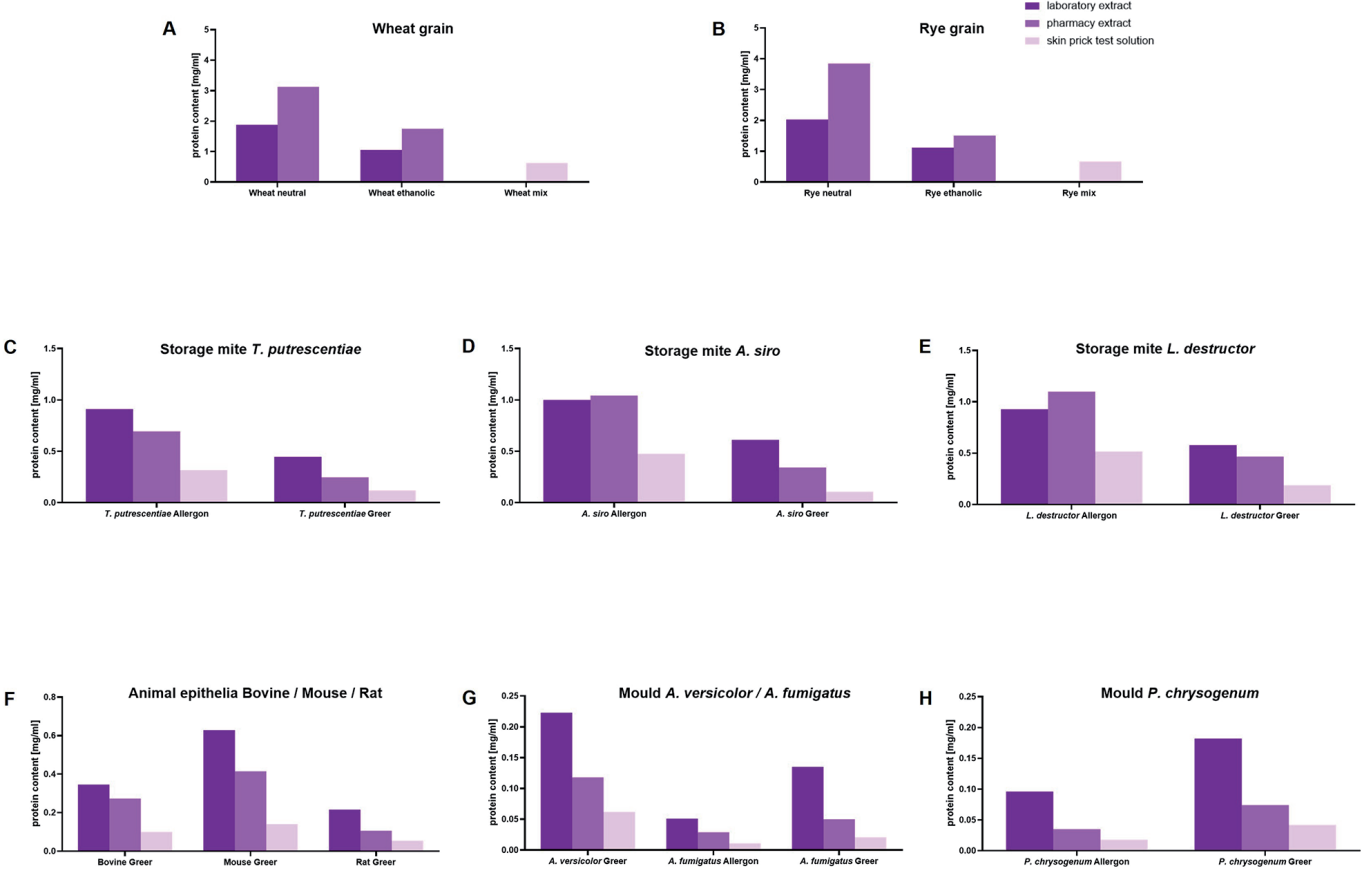
Protein content of the prepared allergen solutions according to laboratory conditions, pharmacy conditions, and in skin prick test solutions from pharmacy extracts. For wheat and rye grain allergen solutions, the neutral and ethanolic fractions as well as a mix of 6 parts neutral and 1 part ethanolic fraction were quantified.

The protein recovery from the allergen source materials used with at least 20 mg substance per mL extraction buffer covered a range of 0.02 – 4 mg/mL (Figure 2). Allergen source materials such as wheat and rye grains per se contain a higher protein content than animal epithelia or molds. However, the amount of allergen source material per extraction volume cannot be increased indefinitely in order to achieve higher protein concentrations in the generated extracts, as otherwise extraction is no longer possible. Mechanical cell disruption and buffer systems can influence the protein recovery, especially for source materials with a low total protein content. As shown in Figure 3, all allergen source materials used were extracted including mechanical disruption and specific buffer (laboratory extract) in comparison to end-over-end rotation in 0.9% NaCl (pharmacy extract). For source material with a low total protein content, such as animal epithelia (Figure 3F) and molds (Figure 3G, H), laboratory extraction procedures increased the protein recovery by up to 200%. Protein extraction from storage mites (Figure 3C, D, E) did not or only slightly benefit from laboratory extraction conditions. For protein-rich source material such as wheat/rye whole grain material (Figure 3A, B), no mechanical cell destruction was intended. Under laboratory conditions, cereal extracts were dialyzed after extraction, which apparently reduced protein recovery by ~ 50%. 

Another aspect that influenced the quantitative protein recovery is that allergen source material from different suppliers was used. For the investigated storage mite source material, starting with the same amount of allergen source material per extraction buffer, ~ 2-fold higher protein concentrations were measured in all allergen extracts when using allergen source material obtained from Allergon compared to material obtained from Stallergens Greer (Figure 3C, D, E). The opposite was found in the case of molds; here only half the protein concentrations were achieved with the Allergon source materials compared to Stallergenes Greer source material (Figure 3G, H). 

### Influence of material and method on protein quality and differences in protein patterns 

**Figure 4:**
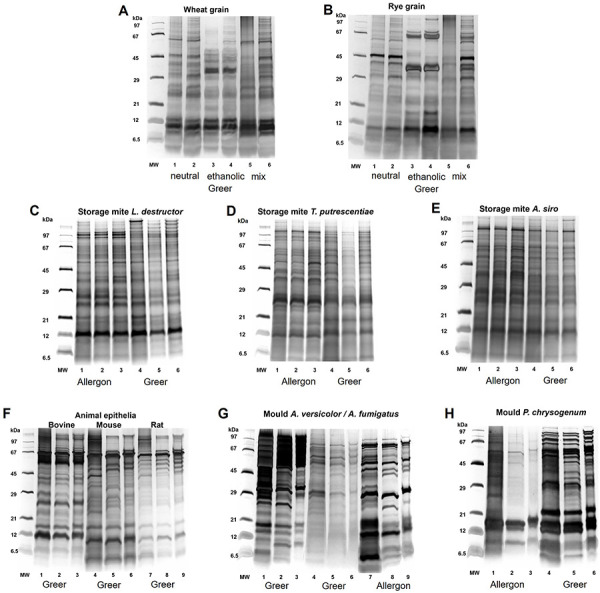
Silver-stained protein patterns after SDS-PAGE of allergen solutions prepared according to laboratory conditions (lanes 1, 4, 7), adapted to pharmacy conditions (lanes 2, 5, 8) and of SPT solutions (mix) generated from pharmacy extracts (lanes 3, 6, 9). The supplier of the allergen sources (Allergon/(Stallergenes) Greer) is indicated below the corresponding gel lanes. For wheat and rye, the laboratory conditions for neutral and ethanolic extracts are shown in lanes 1 and 3, pharmacy conditions in lanes 2 and 4, and SPT solutions in lanes 5 and 6. Same extract volumes were loaded per lane, protein amounts differ between lanes.

In the qualitative protein silver staining, characteristic protein patterns could be identified for all extracted allergen source materials in the molecular weight range of 5 – 100 kDa (Figure 4A – H). For wheat and rye grain extracts, allergen extract qualities comparable to those obtained under optimal laboratory conditions could be produced adapted to pharmacy conditions. The typical protein pattern of neutral wheat grain extracts (Figure 4A) with bands at 10 – 12 kDa and between 25 – 45 plus 65 kDa was observed, whereas ethanolic extracts showed an additional major band at 35 kDa. In rye extracts (Figure 4B), the protein pattern of neutral extracts was focused on the ranges of 8 – 10 kDa and 30 – 45 kDa, in the ethanolic fraction major bands were detected especially at 35 and 65 kDa. The resulting SPT solutions formulated as mix from pharmacy extracts (Figure 4A, B lane 6), prepared in a ratio of six parts neutral extract plus one part ethanolic extract, showed more distinct protein bands (combining characteristic bands from both neutral and ethanolic extraction) than comparable laboratory solutions after dialysis (Figure 4A, B lane 5). 

Prepared storage mite extracts showed homogeneous and comparable protein band patterns independent of the extraction method and the source material used (Figure 4C, D, E). Here, the chosen extraction conditions seemed to predominantly influence the protein quantity, but not the protein quality. All three storage mites showed individually characteristic protein band patterns that can be clearly assigned to the respective species independently of the source material and extraction method used. 

Animal epithelia (Figure 4F) were only available from one allergen supplier, but small differences in the protein patterns were observed for mouse and rat epithelia depending on the extraction method. Here, mechanical cell disruption resulted in additional protein bands of 25 and 43 kDa in the mouse extract (Figure 4F, lane 4) and a 43-kDa band in the rat extract (Figure 4F, lane 7). 

In contrast, the protein patterns of the molds *Aspergillus fumigatus* and *Penicillium chrysogenum* (Figure 4G, H) differed significantly depending on the used source material. Characteristic protein bands for A. fumigatus material from Greer (Figure 4G, lanes 4 – 6) occurred as doublets at molecular weights of 8 – 10 and 22 – 23 kDa, distinct bands at 15, 30, and 60 kDa plus several minor bands in the molecular weight range of 5 – 100 kDa. With A. fumigatus source material from Allergon (Figure 4G, lanes 7 – 9), a similar protein pattern can be seen, but more diffuse, and proteins below 15 kDa appeared very faintly. For *P. chrysogenum*, similar results were obtained for different source material suppliers. Typical protein bands for *P. chrysogenum* extracts can be seen at 15 – 20 kDa and in the molecular weight range of 45 – 80 kDa with source material from Allergon (Figure 4H, lanes 1 – 3), whereas material from Stallergenes Greer (Figure 4H, lanes 4 – 6) shows additional protein bands at 23 and 30 kDa, as well as more defined and numerous protein bands in the molecular weight range of 45 – 100 kDa. 

### From bench to public pharmacy 

**Figure 5:**
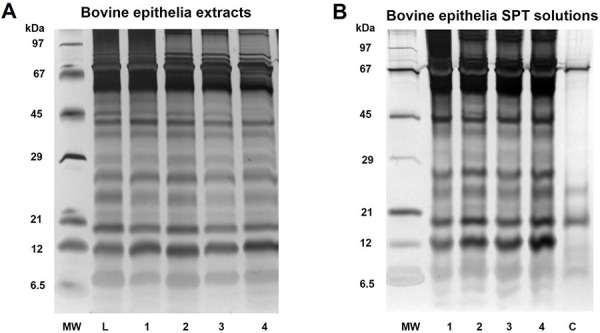
Silver-stained SDS-PAGE of (A) bovine epithelia extracts and (B) bovine epithelia SPT solutions. MW = molecular weight of marker proteins; L = laboratory extract; C = commercial SPT solution; 1 – 4 pharmacy variants I – IV (Table 2A).

In addition to analyzing the conditions for allergen extraction in the laboratory and in the pharmacy, a practical preparation attempt was carried out in a public pharmacy using allergen source material from cattle epithelium as an example. Here, all allergen extracts were prepared in 0.9% NaCl according to Table 2A. The in-process controls carried out on the extract showed pH values of 5 – 6, the extract colors appeared clear and light yellow, and the protein content was between 0.20 and 0.30 mg/mL (Table 2B). Corresponding protein patterns are shown in silver-stained SDS-PAGE (Figure 5A) demonstrating highly reproducible protein profiles with all investigated extraction methods. The procedure for allergen extraction under laboratory conditions with cooling and mechanical cell disruption did not lead to any benefit, neither regarding total protein content nor protein band spectrum (Figure 5A). The reproducibility of bovine epithelial allergen test solutions could be confirmed in practice. Subsequently, sterile SPT solutions were prepared from all pharmacy extract variants (I – IV) and compared with a previously commercially available skin prick solution. Additionally, the protein and allergen (Bos d 2) content was measured in all SPT solutions (Table 2C). The protein content of the commercial cattle SPT solution was about 3-fold lower compared to pharmacy-prepared SPT solutions. However, the allergen content of commercial SPT solution at 4.90 µg/mL was comparable to the pharmacy-prepared SPT solutions at 3.13 – 6.72 µg/mL. The major cattle allergen Bos d 2 can probably be seen at 19 kDa in the silver-stained SDS-PAGE gel (Figure 5B) in all samples. Here, a similar protein pattern was observed in all bovine epithelial SPT solutions, with additional protein bands in the pharmacy-prepared solutions compared to commercial allergen SPT solution. Accordingly, it can be assumed that standardized and qualitatively comparable SPT solutions can be prepared in public pharmacies. 

## Discussion 

The current study has shown that comparable qualities of allergen extracts could be produced under both optimal laboratory and adapted pharmacy conditions. Depending on the intrinsic protein content of the investigated allergen source material, the allergen source materials with low protein content, such as mold and animal epithelia, benefited from mechanical disruption methods that doubled the total protein concentration of the final allergen extracts (Figure 3). Earlier work on mold antigens in airborne filter samples at composting workplaces has showed that the extractable protein content could be significantly increased by mechanical homogenization [14]. Overall, standardization of mold extracts for allergy tests is notoriously difficult, mainly due to the genetically variable source material, as molds have a high mutation frequency, and one fungal species can comprise different strains. In addition, the cultivation and the extraction conditions are another cause for the variability of the resulting fungal allergen extracts [15, 16, 17]. It has been shown that mechanical cell/spore disruption, extraction time and temperature, protease inhibitors, and the ratio of buffer to raw material have an influence on the quality of the fungal extracts. This variability was also observed in the present study using allergen source material obtained from the commercial providers Allergon or Stallergenes Greer for *A. fumigatus* and *P. chrysogenum* (Figure 4G, H). A multicenter study has already investigated that differences in mold allergen solutions may also have an impact on the diagnostic results of SPT regarding test sensitivity and concordance with serological IgE measurement [18, 19]. Furthermore, these studies showed that SPT solutions with a high protein and antigen content exhibited increased test sensitivity. Therefore, source material with a higher protein content will probably be more suitable for the generation of high-quality mold allergen extracts. 

The situation is different for allergen sources with a higher protein content, such as storage mites and wheat and rye grains, which showed no differences depending on the extraction method itself. However, the protein content in laboratory-produced wheat and rye extracts after dialysis was significantly reduced compared to non-dialyzed pharmacy extracts. As dialysis procedures are difficult to implement in public pharmacies, they will currently not be considered in future allergen extract preparations. The protein pattern of wheat and rye displayed water-soluble allergens as well as gliadins and glutenin in the ethanolic fraction. The latter ones were predominantly recognized as food allergens or detected in connection with wheat-dependent exercise-induced anaphylaxis (WDEIA), but may also be relevant in the diagnosis of baker’s asthma [20, 21] and should be included in a SPT solution from public pharmacy to achieve high quality allergen test solutions. The intended mixing ratio of 6 parts neutral cereal extract plus 1 part ethanolic extract plus 50% glycerine results in an ethanol content of ~ 5% or less in the final SPT solution and would therefore not be critical for application to the skin. In allergen extracts of the three storage mites, a doubling of the extractable protein content was measurable when comparing source materials of Allergon with those of Stallergenes Greer (Figure 3). Such effects must be verified in further studies so that the amounts of allergen source material per volume of extraction buffer can be adjusted accordingly in a future standard operation procedure (SOP) for the corresponding source materials. Standardization of allergen test solutions is important and concerns not only occupational allergen extract solutions but also prominent allergen sources like birch pollen [22], peanut extracts [23, 24], and seafood [25], which showed significant differences in individual allergens and immunogenicity depending on the extract preparation. If clinically relevant major allergens are identified and corresponding allergen quantification assays are available, these are valuable tools to detect and standardize the allergen amount in the end products [26]. For most occupational allergens, however, not only one major allergen is relevant, but several allergens are involved in IgE-mediated reactions. However, if there are no major allergens, other parameters must be used for quality validation. For SPT solutions, an EAACI position paper on the standardization of occupational allergen test solutions [27] recommended the determination of protein and antigen content to increase test sensitivity. In the current study, only protein concentrations and protein patterns are presented (in concordance with batch release requirements for commercial SPTs of rare allergen sources), but during allergen extract preparation under adapted pharmacy conditions antigen levels were exploratively determined (data not shown), which correlated well with the measured protein concentrations. 

A first transfer experiment from the laboratory bench side to the public pharmacy practice was conducted with bovine epithelia, as these displayed a characteristic protein profile with an estimated mean protein content of 0.2 – 0.4 mg/mL. The allergen extracts and SPT solutions were produced exclusively with materials that can be used in accordance with the pharmacy operating regulations under the most sterile conditions possible by a pharmaceutical technician according to the specified protocol and formulation of a public pharmacy. All in-process and additional laboratory-based testing of the final products showed highly comparable protein profiles of good quality (Figure 5A, B). Even though mechanical disruption in extract variant I and II showed slight increases in the protein concentration of extract, this effect was neither confirmed in the final SPT solution nor in Bos d 2 allergen concentration (Table 2C). The highest Bos d 2 content was measured in extraction variants II and IV with more than 6 µg/mL, which might be due to longer extraction times in these variants, or the inhomogeneous source material, containing both hair and epithelia, was responsible for the differences. Nevertheless, all bovine allergen solutions prepared in the pharmacy had a Bos d 2 content comparable to commercial skin test solution of 4.9 µg/mL (Table 2C). Based on these results and for reasons of practicability, pharmacy variant IV, extraction for 2 hours by end-over-end rotation, was selected for all further SPT preparations. Another important aspect in the preparation of SPT solutions in public pharmacies is the identity control performed on the used allergen source material [7]. Since each allergen source exhibited an individual protein pattern (Figure 4) which was reproducible under both laboratory and pharmacy extraction conditions (Figure 5), SDS-PAGE combined with silver staining could serve as an identity control as well as a possible end product control if the method can be performed in the pharmacy. 

Until now, extraction methods for standardized allergen solutions from different allergen materials (wheat and rye grains, storage mites, animal dander, and molds) prepared under conditions of a public pharmacy have been evaluated. The next steps for a standardized and validated allergen extraction procedure are to lay down the proceedings in SOPs which comply to requirements of the German Medicinal Products Act (AMG), Pharmacy Operating Ordinance (ApBetrO), and Ph. Eur. and to implement the practical application of SOPs in pharmacy practice to enable the independent production of high-quality allergen extracts. 

## Acknowledgment 

We are thankful to Frank Führer (Paul-Ehrlich-Institut) for valuable and constructive discussion. 

## Disclaimer 

RJ, SSchü, KPT, AB, VM: The views expressed in this manuscript are the personal views of the authors and may not be understood or quoted as being made on behalf of or reflecting the position of the respective national competent authority, the European Medicines Agency, or one of its committees, or the German Federal Ministry of Health. 

## Authors’ contributions 

Conception and design of the study: Who was responsible for the idea and design of the study? MR, SK, VM, KEPT, RJ. Data collection: Who collected the data or conducted the experiments? SM, SK. Data analysis and interpretation: Who analyzed the data and interpreted the results? SK, RJ, SM, LMA, IS, SS, AB, TK, VM, MR. Manuscript drafting: Who wrote the manuscript and who reviewed it? SK, RJ, SM, LMA, IS, SS, KEPT, AB, TK, VM, MR. 

## Funding 

This study was financially supported by the German Social Accident Insurance (DGUV) (part of the IPA-project IPA 164-Berufsderma and Research support FB317A (Quality control of the diagnostics for occupational type I allergies) granted to Paul-Ehrlich-Institut). The funder had no role in study design, data collection and analysis, decision to publish, or preparation of the manuscript. 

## Conflict of interest 

The authors declare that the research was conducted in the absence of any commercial or financial relationships that could be construed as a potential conflict of interest. Some authors received fees, which are listed below. 

MR received fees for lectures from the following companies and associations between 2020 and 2024: Alk-Abelló Arzneimittel GmbH, Berufsverband Deutscher Baubiologen VDB e.V, Haus der Technik, LetiPharma, ThermoFisher Scientific (Phadia). With regard to the content of this article, there are no conflicts of interest that could arise from an employment relationship, benefits for lectures, or other activities. 

**Table 1.** Commercially allergen source materials plus the respective allergen quantities used for allergen extract preparation.

**Allergen source**	**Allergen material & provider**	**Lot No.**	**Weight/volume**
Wheat grain	Stallergenes Greer, Wheat, whole *Triticum aestivum*	358419-3	250 mg/mL
Rye grain	Stallergenes Greer, Rye, *Secale cereal*	254738-2	250 mg/mL
*Lepidoglyphus destructor*	Allergon, semipurified mites	498518002	20 mg/mL
*Lepidoglyphus destructor*	Stallergenes Greer, natural mite	P6100894	20 mg/mL
*Tyrophagus putrescentiae*	Allergon, semipurified mites	499618301	20 mg/mL
*Tyrophagus putrescentiae*	Stallergenes Greer, natural mite	P6676667	20 mg/mL
*Acarus siro*	Allergon, semipurified mites,	497618302	20 mg/mL
*Acarus siro*	Stallergenes Greer, natural mite	P6676664	20 mg/mL
Cattle epithelia	Stallergenes Greer, Cattle epithelia, *Bos taurus*, Acetone powedered epithelia	366791	20 mg/mL
Mouse epithelia	Stallergenes Greer, mouse epithelia, *Mus musculus*, Acetone powedered epithelia	374275	20 mg/mL
Rat epithelia	Stallergenes Greer, rat epithelia, *Rattus norvegicus*, Acetone powedered epithelia	374806	20 mg/mL
*Aspergillus versicolor*	Stallergenes Greer er, *Aspergillus versicolor*	301774-1	25 mg/mL
*Aspergillus fumigatus *	Allergon, *Aspergillus fumigatus*	100919032	25 mg/mL
*Aspergillus fumigatus *	Stallergenes Greer, *Aspergillus fumigatus*	77704	25 mg/mL
*Penicillium chrysogenum*	Allergon, *Penicillium chrysogenum*	109018011	25 mg/mL
*Penicillium chrysogenum*	Stallergenes Greer, *Penicillium chrysogenum*	351234	25 mg/mL

**Table 2A.** Cattle epithelia allergen solutions prepared with different extraction methods (I – IV) in a public pharmacy.

**Extraction variant**	**Roll mixer (10 min. at 36 rpm, RT)**	**Potter (5 min. at 2,000 rpm, cooling)**	**TOPITEC (10 min. at 2,500 rpm, RT)**	**End over end rotation, (2 h, 18 rpm, RT)**	**Ultra sonic bath (10 min. in iced water)**	**Centrifugation**
Laboratory	x	x	–	–	x	4 °C
Pharmacy I	x	x	–	–	x	RT
Pharmacy II	x	–	x	–	x	RT
Pharmacy III	x	x	–	–	–	RT
Pharmacy IV	–	–	–	x	–	RT

**Table 2B.** In-process control and total protein content of the cattle epithelial allergen extracts produced in a laboratory and in a public pharmacy.

**Extraction variant**	**Extract pH**	**Extract color**	**Protein conc. (µg/mL)**
Laboratory	5 – 6	Clear, light yellow	240
Pharmacy I	5 – 6	Clear, light yellow	303
Pharmacy II	5 – 6	Clear, light yellow	237
Pharmacy III	5 – 6	Clear, light yellow	201
Pharmacy IV	5 – 6	Clear, light yellow	242

**Table 2C:** Protein and Bos d 2 allergen content of prepared SPT solutions.

**SPT-solution based on extract variant**	**Protein conc. (µg/mL)**	**Bos d 2 conc. (µg/mL)**
Pharmacy I	121	4.6
Pharmacy II	117	6.7
Pharmacy III	104	3.1
Pharmacy IV	113	6.4
Commercial SPT supplier	39	4.9
